# Identification of long non-coding RNA-related and –coexpressed mRNA biomarkers for hepatocellular carcinoma

**DOI:** 10.1186/s12920-019-0472-0

**Published:** 2019-01-31

**Authors:** Fan Zhang, Linda Ding, Li Cui, Robert Barber, Bin Deng

**Affiliations:** 1 Vermont Genetics Network, University of Vermont, Burlington, VT 05405 USA; 2 Department of Biology, University of Vermont, Burlington, VT 05405 USA; 3 School of Medicine, University of California, San Diego, 9500 Gilman Drive, La Jolla, CA 92093-0606 USA; 4 Department of Neurosciences, School of Medicine, University of California, San Diego, 9500 Gilman Drive #0949, La Jolla, CA 92093 USA; 5 Department of Pharmacology and Neuroscience, University of North Texas Health Science Center, Fort Worth, TX USA

**Keywords:** Long non-coding RNA, Biomarker discovery, Hepatocellular carcinoma

## Abstract

**Background:**

While changes in mRNA expression during tumorigenesis have been used widely as molecular biomarkers for the diagnosis of a number of cancers, the approach has limitations. For example, traditional methods do not consider the regulatory and positional relationship between mRNA and lncRNA. The latter has been largely shown to possess tumor suppressive or oncogenic properties. The combined analysis of mRNA and lncRNA is likely to facilitate the identification of biomarkers with higher confidence.

**Results:**

Therefore, we have developed an lncRNA-related method to identify traditional mRNA biomarkers. First we identified mRNAs that are differentially expressed in Hepatocellular Carcinoma (HCC) by comparing cancer and matched adjacent non-tumorous liver tissues. Then, we performed mRNA-lncRNA relationship and coexpression analysis and obtained 41 lncRNA-related and -coexpressed mRNA biomarkers. Next, we performed network analysis, gene ontology analysis and pathway analysis to unravel the functional roles and molecular mechanisms of these lncRNA-related and -coexpressed mRNA biomarkers. Finally, we validated the prediction and performance of the 41 lncRNA-related and -coexpressed mRNA biomarkers using Support Vector Machine model with five-fold cross-validation in an independent HCC dataset from RNA-seq.

**Conclusions:**

Our results suggested that mRNAs expression profiles coexpressed with positionally related lncRNAs can provide important insights into early diagnosis and specific targeted gene therapy of HCC.

## Background

Hepatocellular carcinoma (HCC) is a highly prevalent, treatment-resistant malignancy with a multifaceted molecular pathogenesis and is also one of the most common cancers and the third most common cause of death by cancer worldwide [1]. Geographic areas with the highest prevalence are located in Africa and Eastern Asia, likely due to the increasing prevalence of chronic hepatitis B or C. The incidence of HCC is also rising in the US. According to the American Cancer Society, an estimated 39,230 people were diagnosed with HCC in the United States during 2016 and about 27,170 people will die of the cancer [2].

Although significant effort has been directed toward the improvement of surgical and medical treatment, the prognosis for patients with advanced stages of HCC remains poor [3]. To improve diagnosis and treatment efficiency, a further understanding of molecular mechanisms of HCC progression is urgently needed.

Several research strategies, high-throughput genomic microarray in particular, have been used to investigate the molecular characteristics of HCC. Many molecular biomarkers with aberrant expression have been identified in HCC tissues, including NDRG1, Importin-α1, FOXP1, and PTPN12. Although these findings have greatly improved diagnostic and therapeutic strategies, some limitations remain. First, because of the large number of mRNA and proteins in the profiles, specific targets are difficult to identify. Second, changes at the mRNA level are not always consistent with those at the protein level, suggesting that a high level of background noise may exist. Third, traditional microarray methods identify mRNAs that are differentially expressed between normal vs cancer, but don’t consider the relationship between coding biomarkers and non-coding RNA, which are likely to be etiologically important.

Long noncoding RNAs (lncRNAs) are a class of noncoding RNA transcripts longer than 200 nucleotides that were previously believed to represent transcriptional noise. However, they have recently been identified as functional molecules. Emerging studies show that lncRNA play important roles in basic biology, ranging from transcriptional and post-transcriptional regulation to the control of cell cycle distribution, imprinting control, cell differentiation and tumorigenesis. Several lncRNAs have been reported to regulate the development of HCC and targets include HOXA transcription at the distal tip (HOTTIP) [4, 5], MEG3 maternally expressed 3 (MEG3) [6–8], highly upregulated in liver cancer (HULC) [9, 10], and ZNFX1 Antisense RNA 1 (ZFAS1) [11–13].

We hypothesized that combining mRNA expression profiles from traditional microarray methods and lncRNA expression profiles from lncRNA arrays may help identify a subset of candidate lncRNA-related and -coexpressed mRNA biomarkers with higher confidence and reliability. We defined lncRNA-related and -coexpressed mRNA biomarkers as mRNA biomarkers that are coexpressed with and have positional relationships with adjacent lncRNAs. We selected mRNA biomarkers based on two criteria: 1) differential expression between normal and cancer tissue samples; 2) a positional relationship to lncRNAs that are differentially expressed between normal and cancer samples.

In this paper, we first identify 3543 mRNA biomarkers differentially expressed between normal and cancer samples using t statistics and qvalues. Then we overlapped these transcripts with the results from lncRNA arrays to identify 41 lncRNA-related and -coexpressed mRNA biomarkers. Next, we performed network analysis, functional analysis and pathway analysis for the 41 lncRNA-associated mRNA biomarkers. Finally, we used an independent dataset and a SVM model to validate the prediction performance of the 41 identified lncRNA-related and -coexpressed mRNA biomarkers. Our results showed that combined microarray and lncRNA analysis improved biomarker discovery for the treatment of HCC.

## Methods

### lncRNA and mRNA microarray expression profiling

We downloaded Arraystar human lncRNA and mRNA microarray data for a cohort of 16 Human HCC samples and 16 adjacent non-tumor samples from GEO (GSE58043, GSE89186, GSE64631, and GSE55191, http://www.ncbi.nlm.nih.gov/geo) [14–16]. The Arraystar human lncRNA and mRNA microarray data included five positional relationship between lncRNA and mRNA: 1) “exon sense-overlapping”: the lncRNA’s exon is overlapping a coding transcript exon on the same genomic strand; 2) “intron sense-overlapping”: the lncRNA is overlapping the intron of a coding transcript on the same genomic strand; 3) “intronic antisense”: the lncRNA is overlapping the intron of a coding transcript on the antisense strand; 4) “natural antisense”: the lncRNA is transcribed from the antisense strand and overlapping with a coding transcript; and 5) “bidirectional”: the lncRNA is oriented head to head to a coding transcript within 1000 bp. If the Arraystar human lncRNA and mRNA microarray data showed no overlapping or bidirectional coding transcripts nearby the lncRNA, we defined the relationship between mRNA and lncRNA as “intergenic”.

### Statistical analysis

We first performed a Box-Cox Power Transformation [17] using a powerTransform function (car package in R 3.4.0) to make the distribution of each mRNA and lncRNA in each sample approximately normal.

We used a two-sample, two-sided t-test [18, 19] to determine whether there was no difference between the mean of gene expression in HCC samples and that in normal samples. The null hypotheses was

1 $$ {H}_0:{\mu}_T={\mu}_N $$

where *μ*_*T*_ is the mean of gene expression in HCC samples, and *μ*_*N*_ is the mean of gene expression in normal samples. This null hypothesis was tested against the following alternative hypothesis:

2 $$ {H}_1:{\mu}_T\ne {\mu}_N $$

*P* values were determined by Welch’s t-test. Qvalues were adjusted for false discovery rate control using *qvalue* package from Bioconductor.

### Pathway analysis

Network analyses were generated through the use of Ingenuity Pathway Analysis (IPA, Redwood City, CA, USA). The top scoring network of interactions was presented for the concurrent under-expressed and the concurrent over-expressed genes. This software analyzes molecular signatures in the context of known biological response and regulatory networks as well as canonical pathways.

DAVID functional analyses were used to identify biological functions that were most significantly enriched with expression changes [20].

Interpretation of biological pathways was conducted with the database we developed: Integrated Pathway Analysis Database (IPAD) (http://fzhang.w3.uvm.edu/ipad/) [21]. The enrichment scores used to select significant pathways were defined by *p*-value.

### Performance measurement

We used the following five measurements for our evaluation: (1) Sensitivity (2) Specificity, (3) Precision, (4) Accuracy, and (5) Area Under the Curve.

$$ Sensitivity=\frac{true\ positive}{true\ positive+ false\ negative} $$

$$ Specificity=\frac{true\ negative}{true\ positive+ false\ positive} $$

$$ Precision=\frac{true\ positive}{true\ positive+ false\ positive} $$

$$ Accuracy=\frac{true\ positive+ true\ negative}{true\ positive+ true\ negative+ false\ positive+ false\ negative} $$

## Results

The Arraystar Human lncRNA and mRNA microarray profiles contained two states (tumor vs. non-tumor) with 16 samples corresponding to each state. We obtained 3543 significantly differentially expressed mRNA biomarkers (mapped to 1932 genes) with qvalue < 0.05, among which 2066 (946 genes) were over-expressed and 1477 (986 genes) were under-expressed in HCC tumor.

**Fig. 1:**
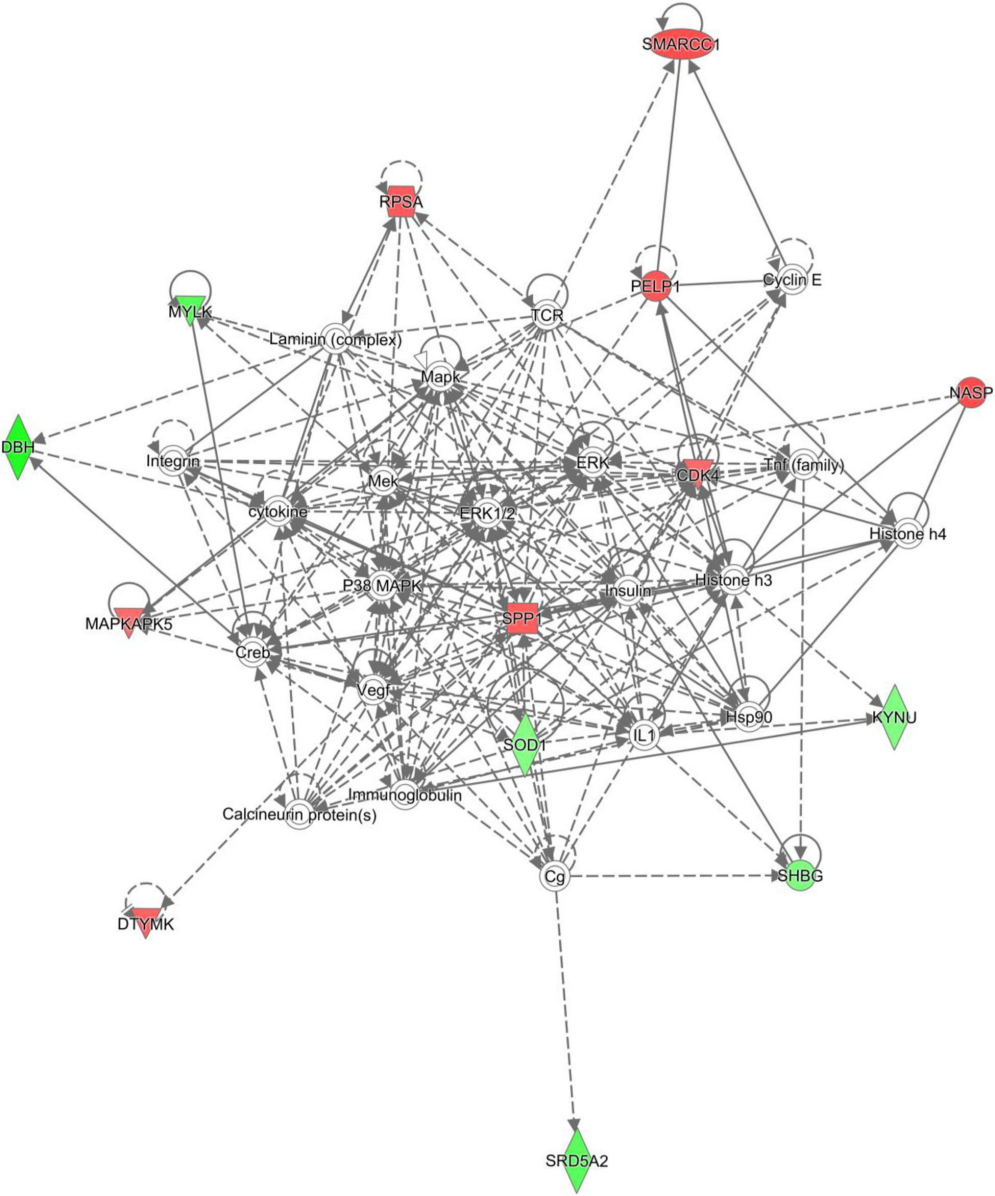
The 41 mRNAs biomarkers involved in Endocrine System Development and Function, Molecular Transport, Small Molecule Biochemistry. Red stands for over-expressed and green for under-expressed

**Fig. 2:**
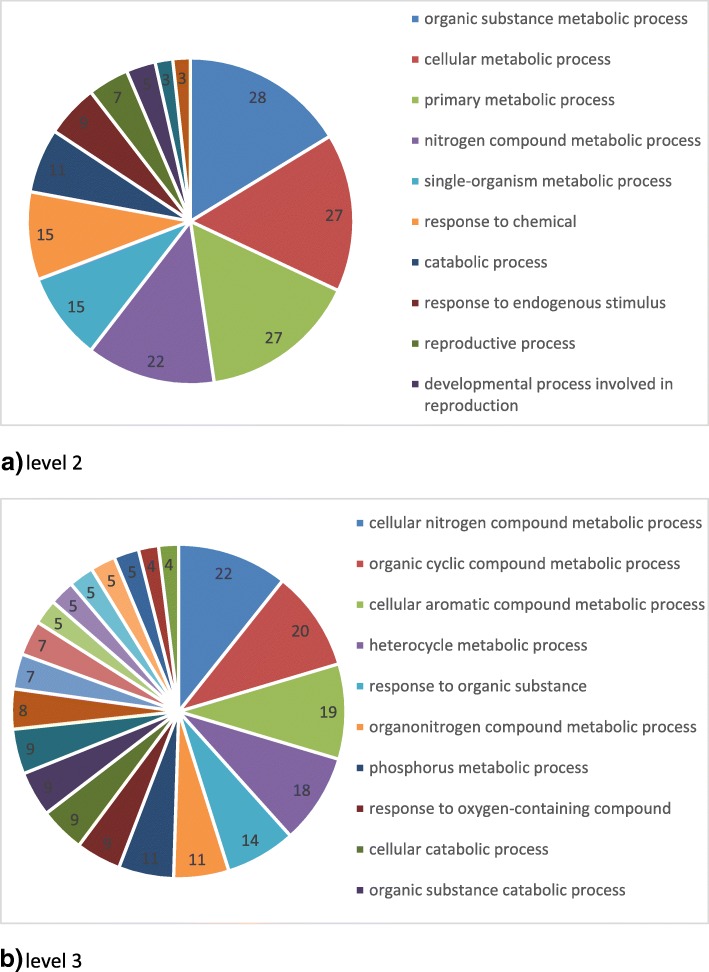
Gene ontology biological processes enrichment analysis for 41 mRNA biomarkers. The GO terms were categorized into (**a**) biological processes at level 2 and (**b**) biological processes at level 3

Forty one mRNA biomarkers (Table 1) met our criteria of (1) being differentially expressed between 16 normal and 16 cancer samples with qvalue < 0.05; and 2) being positionally related to lncRNA which were differentially expressed between 16 normal and 16 cancer samples with qvalue < 0.05. The information corresponding to the positional relationships of mRNA/miRNA and lncRNAs were identified to predict the role of lncRNAs in regulating nearby genes. The positional relationship included exon sense-overlapping (7), natural antisense (16), bidirectional (9), intronic antisense(6), intron sense-overlapping (2), and sense overlap (1) (Table 2). Network analyses were performed with Ingenuity Pathway Analysis and the top four networks were identified (Table 3 and Fig. 1). We identified four networks: 1) Endocrine System Development and Function, Molecular Transport, Small Molecule Biochemistry; 2) Immunological Disease, Inflammatory Disease, Inflammatory Response; 3) Amino Acid Metabolism, Molecular Transport, Small Molecule Biochemistry; and 4) Metabolic Disease, Developmental Disorder, Hereditary Disorder. Gene ontology analysis with DAVID described the biological processes of the 41 mRNA biomarkers (Fig. 2a and b). Pathway analysis were generated using the IPAD [21] (Table 4). Pathways linked with the 41 mRNA biomarkers included Metabolism, Hemostasis, Cell Cycle, Signaling, Disease, Immune system, and Gene Expression, which are consistent with previous results we found [22–25].

**Table 1:** Forty one mRNA biomarkers with relationship to coexpressed lncRNAs (qvalue< 0.05 for mRNA and qvalue < 0.05 for lncRNA)

mRNA	Gene	Gene expression	mRNA qvalue	lncRNA Probe name	relationship	lncRNA expression	lncRNA qvalue
NM_000787	DBH	down	0.0153	ASHG19A3A038177	natural antisense	down	0.0317
NM_015987	HEBP1	down	0.0153	ASHG19A3A048399	bidirectional	down	0.0252
NM_001172440	ENDOU	down	0.0161	ASHG19A3A055103	intronic antisense	down	0.0285
NM_001130997	FAM58A	up	0.0170	ASHG19A3A041726	bidirectional	up	0.0331
NM_000075	CDK4	up	0.0216	ASHG19A3A048765	natural antisense	up	0.0396
NM_000744	CHRNA4	down	0.0218	ASHG19A3A018571	intronic antisense	down	0.0285
NM_003074	SMARCC1	up	0.0231	ASHG19A3A022584	intronic antisense	up	0.0285
NM_025139	ARMC9	up	0.0240	ASHG19A3L0001156	sense overlap	up	0.0472
NM_014053	FLVCR1	up	0.0270	ASHG19A3A007495	bidirectional	up	0.0479
NM_000348	SRD5A2	down	0.0271	ASHG19A3L0001181	exon sense-overlapping	down	0.0357
NM_001012321	RPSA	up	0.0279	ASHG19A3A020907	natural antisense	down	0.0293
NM_001334	CTSO	down	0.0284	ASHG19A3A026206	natural antisense	down	0.0415
NM_030789	HM13	up	0.0284	ASHG19A3A017537	intronic antisense	down	0.0479
NM_000454	SOD1	down	0.0311	ASHG19A3A018779	bidirectional	down	0.0468
NM_002394	SLC3A2	up	0.0314	ASHG19A3A000043	bidirectional	up	0.0291
NM_144778	MBNL2	down	0.0315	ASHG19A3L0000699	exon sense-overlapping	down	0.0439
NM_001146279	SHBG	down	0.0315	ASHG19A3A007528	exon sense-overlapping	down	0.0380
NM_003631	PARG	up	0.0328	ASHG19A3A043936	intronic antisense	up	0.0289
NM_000182	HADHA	down	0.0330	ASHG19A3A015417	natural antisense	up	0.0489
NM_003668	MAPKAPK5	up	0.0333	ASHG19A3A055106	natural antisense	up	0.0364
NM_016065	MRPS16	up	0.0338	ASHG19A3A044109	bidirectional	up	0.0362
NM_053031	MYLK	down	0.0338	ASHG19A3A023105	intronic antisense	up	0.0285
NM_001040058	SPP1	up	0.0355	ASHG19A3A024471	natural antisense	up	0.0356
NM_145697	NUF2	up	0.0355	ASHG19A3A054586	bidirectional	up	0.0412
NM_172250	MMAA	down	0.0372	ASHG19A3A024820	natural antisense	down	0.0409
NM_001003789	RABL2B	down	0.0378	ASHG19A3A020631	bidirectional	up	0.0252
NM_001040060	SPP1	up	0.0378	ASHG19A3A024471	natural antisense	up	0.0356
NM_207304	MBNL2	down	0.0379	ASHG19A3L0000699	exon sense-overlapping	down	0.0439
NM_020791	TAOK1	up	0.0382	ASHG19A3A009329	exon sense-overlapping	up	0.0412
NM_016632	ARL17A	up	0.0395	ASHG19A3A008470	intron sense-overlapping	up	0.0252
NM_014583	LMCD1	up	0.0397	ASHG19A3A020672	bidirectional	down	0.0311
NM_003937	KYNU	down	0.0405	ASHG19A3A014435	natural antisense	down	0.0252
NM_000582	SPP1	up	0.0418	ASHG19A3A024471	natural antisense	up	0.0356
NM_014389	PELP1	up	0.0419	ASHG19A3A008987	natural antisense	up	0.0437
NM_148921	EPN2	down	0.0433	ASHG19A3A008042	natural antisense	up	0.0329
NM_001165031	DTYMK	up	0.0442	ASHG19A3A007748	exon sense-overlapping	up	0.0285
NM_002482	NASP	up	0.0448	ASHG19A3A044925	natural antisense	up	0.0489
NM_000128	F11	down	0.0449	ASHG19A3A025095	natural antisense	down	0.0252
NM_002022	FMO4	down	0.0468	ASHG19A3A034907	intron sense-overlapping	down	0.0446
NM_001127603	NMRK1	down	0.0470	ASHG19A3A037588	exon sense-overlapping	down	0.0331
NM_003889	NR1I2	down	0.0471	ASHG19A3A021464	natural antisense	down	0.0291

**Table 2:** Statistics for relationships

Relationship	Counts
Bidirectional	9
Exon sense-overlapping	7
Intron sense-overlapping	2
Intronic antisense	6
Natural antisense	16
Sense overlap	1

**Table 3:** Top networks involved

Top Diseases and Functions	Score	Focus Molecules
Endocrine System Development and Function, Molecular Transport, Small Molecule Biochemistry	32	14
Immunological Disease, Inflammatory Disease, Inflammatory Response	32	14
Amino Acid Metabolism, Molecular Transport, Small Molecule Biochemistry	21	10
Metabolic Disease, Developmental Disorder, Hereditary Disorder	3	1

**Table 4:** Pathway analysis for the 41 mRNA biomarkers

Pathway ID	Pathway Name	Molecule	AE
1,430,728	Metabolism	DTYMK;DBH;HADHA;KYNU;SRD5A2	5
109,582	Hemostasis	F11;SOD1;SLC3A2	3
1,640,170	Cell Cycle	CDK4;NUF2;TAOK1	3
69,278	Cell Cycle, Mitotic	CDK4;NUF2;TAOK1	3
74,160	Gene Expression	NR1I2;RPSA;F11	3
162,582	Signal Transduction	HEBP1;SPP1	2
1,643,685	Disease	SPP1;RPSA	2
200,050	Calcineurin-regulated NFAT-dependent transcription in lymphocytes	CDK4;SLC3A2	2
200,170	Nongenotropic Androgen signaling	SHBG;PELP1	2
212,436	Generic Transcription Pathway	NR1I2;F11	2
382,551	Transmembrane transport of small molecules	FLVCR1;SLC3A2	2
453,277	Mitotic M-M/G1 phases	NUF2;TAOK1	2
556,833	Metabolism of lipids and lipoproteins	HADHA;SRD5A2	2
68,877	Mitotic Prometaphase	NUF2;TAOK1	2
68,886	M Phase	NUF2;TAOK1	2
69,306	DNA Replication	NUF2;TAOK1	2
71,291	Metabolism of amino acids and derivatives	DBH;KYNU	2
hsa00380	Tryptophan metabolism	KYNU;HADHA	2
hsa04010	MAPK signaling pathway	MAPKAPK5;TAOK1	2
hsa04142	Lysosome	CTSO;CTSO	2
hsa04510	Focal adhesion	SPP1;MYLK	2
hsa04620	Toll-like receptor signaling pathway	SPP1;CTSO	2

In order to validate the 41 mRNA biomarkers, we built a five-fold cross-validation Support Vector Machine (SVM) model based on all the 32 samples using a radius basis function kernels function. We achieved high prediction performance (AUC = 0.996, precision = 100%, accuracy = 96.9%, sensitivity = 93.8%, specificity = 100%). Further we randomly divided the 16 HCC samples and 16 Normal samples into two groups: training set and testing set. Each group contains 8 HCC samples and 8 Normal samples. We used the testing test to assess the performance of the trained 41 mRNA biomarkers. The testing set was blind and no data from the testing set were used for identification of the 41 mRNA biomarkers and development of the SVM model. We obtained high performances: for the training set (AUC = 1.0, precision = 100%, accuracy = 93.8%, sensitivity = 87.5%, specificity = 100%) and for testing set (AUC = 0.984, precision = 100%, accuracy = 93.8%, sensitivity = 87.5%, specificity = 100%) (Table 5).

**Table 5:** Validation with SVM for the 41 mRNA biomarkers

Predicted	Training set		Testing set		RNA Testing set	
	HCC	Normal	HCC	Normal	HCC	Normal
HCC	7	0	7	0	15	3
normal	1	8	1	8	6	18
Precision	100%		100%		83.3%	
Accuracy	93.8%		93.8%		78.6%	
Sensitivity	87.5%		87.5%		71.4%	
Specificity	100%		100%		85.7%	
AUC	1		0.984		0.824	

In order to validate the prediction performance of the 41 mRNA biomarkers for cross-platform dataset, we downloaded from GEO a RNA-seq dataset (GSE94660 [26]) which contains 21 HCC samples and 21 Non-neoplastic liver samples. We used the 38 genes as variables. The gene expression data in training microarray first was averaged by the 38 genes and then was normalized to 0–1 range by a min-max transformation function: y = (x-min)/(max-min). After normalization, a SVM model with five-fold cross-validation was used for learning the training set. The Reads Per Kilobase Million (RPKM) data in testing RNA-seq containing the 38 genes was normalized using the min-max transformation and used as blind testing set. The SVM model achieved high performances (AUC = 0.824, precision = 83.3%, accuracy = 78.6%, sensitivity = 71.4%, specificity = 85.7%). The results showed that lncRNA-related and -coexpressed mRNA biomarkers had high prediction accuracy within the training and testing sets.

## Discussion

The top four networks we identified were similar to previously reported results [27–29]. For example, De et al. used high-density oligoarrays to identify consistent differences in gene-expression between HCC and normal liver tissue. Their network analysis of differentially expressed genes classified cellular and biological functions related to regulation of gene expression and post-translational modification in HCV-related primary HCC. These included Cellular Growth and Proliferation and Cell-To-Cell Signaling and Interaction in HCV-related non HCC samples; Cellular Growth and Proliferation and Cell Cycle in metastasis [30]. Xu et al. combined Chromatin immunoprecipitation (ChIP) on chip along with gene expression microarrays to create a genome-wide scale map of TFCP2 targets as well as the molecular function and pathways regulated by TFCP2 in HCC. They found that TFCP2-ChIP targets in SK-HEP-1 were functionally associated with cancer, cell movement, cell cycle, cell-to-cell signaling and interaction, cellular growth and proliferation [28]. Das et al. performed gene expression profiling between two groups of patients with HCV: one with HCC recurrence and second without recurrent HCC and revealed 194 differentially regulated genes between the two groups. They found that under-expressed genes were associated not only with HCC recurrence, but also with regulation of the innate immune response, cell-to-cell signaling and interaction, and the inflammatory response [29].

The Signaling, Disease, Metabolism, Cell Cycle, Immune system, and Gene Expression pathways linked with the 41 mRNA biomarkers were also reported in previous findings [22–25]. For example, two main pathogenic mechanisms were involved during hepatocarcinogenesis: (1) cirrhosis associated with hepatic regeneration after tissue damage caused by hepatitis infection, toxins or metabolic influences, and (2) mutations occurring in single or multiple oncogenes or tumor suppressor genes. Both mechanisms were linked in several important cellular signaling pathways. These signal pathways are of interest from a therapeutic perspective, because targeting them might help to reverse, delay or prevent tumorigenesis [24]. Numerous signaling modules including some related to growth factor signaling (e.g., IGF, EGF, PDGF, FGF, HGF), cell differentiation (WNT, Hedgehog, Notch), and angiogenesis (VEGF) have become a major source of targets for novel therapies in HCC. Different molecular mechanisms have been shown to induce aberrant pathway activation, such as point mutations, chromosomal aberrations, and epigenetically driven down-regulation [25]. Huang et al. investigated the role of EGF-EGFR signaling pathway in the development of human hepatocellular carcinoma (HCC) inflammatory environment by measuring the gene profiles of inflammatory cytokines from HCC. They found that HCC proliferation, metastasis and production of inflammatory cytokines were regulated via EGF-EGFR signaling pathways, which represent potential therapeutic targets for HCC [23].

Some biomarkers and their association with HCC already have been reported. For example, CDK4 (Cyclin Dependent Kinase 4) has been implicated in a number of cancer types. Jin et al. demonstrated the activation of cdk4 triggers and inhibitors of cdk4 for the prevention/treatment of Non-alcoholic Fatty Liver Disease [31]. Secreted phosphoprotein-1 (SPP1) was found to be overexpressed in metastatic hepatocellular carcinoma (HCC), and had potential to act as both a diagnostic marker and a therapeutic target for HCC [32]. Li et al. found that downregulation of Superoxide Dismutase 1 (SOD1) was correlated with histopathological grading and might be a good candidate gene for HCC [33].

LncRNA/mRNA expression profiling has been widely used for biomarker discovery of cancers, for example, liver cancer, gastric cancer, bladder cancer, colon cancer, pancreatic cancer, laryngeal cancer, and colorectal cancer. This is because LncRNAs is important in identifying biomarkers for various human cancers. Unraveling the co-expression pattern between mRNAs and lncRNAs can further help researchers better understand the mechanism of various human cancers. The lncRNA-related and -coexpressed method to detect mRNA biomarkers we presented in the study can work not only for HCC but also for all other human cancers.

The advantage of the lncRNA-related and -coexpressed method to detect mRNA biomarkers is that we consider the co-expression between mRNA and lncRNA, filter out some unimportant mRNAs and lncRNAs by setting significant threshold, and focus on the most important mRNAs and lncRNAs and their coexpressed networks. Compared with the original findings from the datasets (GSE58043, GSE89186, GSE64631, and GSE55191, http://www.ncbi.nlm.nih.gov/geo) [14–16], it shows that we found the Endocrine System Development and Function as top network associated with liver cancer. This finding is consistent with the fact that liver abnormalities have strong association with endocrine diseases [34, 35]. It would be difficult to discover the Endocrine System Development and Function as top network without using lncRNAs’ coexpression as one kind of filter. As shown in the result section, there are 3543 significantly differentially expressed mRNA biomarkers at first. It is the lncRNA coexpression filter in our method that shortens the number of biomarker candidates and unravels the final 41 mRNAs (38 genes) which functionally link to the Endocrine System Development and Function.

There is a limitation of the study with small sample size. We have tried our best to collect all the four available LncRNA/mRNA expression profiling related to HCC [14–16]. In the future, we will continue to collect HCC LncRNA/mRNA expression profiling data with our collaborators.

## Conclusion

We developed a lncRNA-related and -coexpressed method to detect mRNA biomarkers with HCC. Top networks such as “Endocrine System Development and Function, Molecular Transport, Small Molecule Biochemistry” and enriched pathways such as Cell Cycle, Signaling, Metabolism, and Immune System were also discovered. Unraveling these intricate networks and pathways is essential to understanding the biological mechanisms of HCC development and progression. Our method has the potential to provide a basis for biomarker identification in HCC or other diseases.

## Abbreviation

AUC: Area Under the Curve
ChIP: Chromatin immunoprecipitation
DAVID: the Database for Annotation, Visualization and Integrated Discovery
HCC: Hepatocellular Carcinoma
IPA: Ingenuity Pathway Analysis
IPAD: Integrated Pathway Analysis Database
lncRNA: Long noncoding RNA
RPKM: The Reads Per Kilobase Million
SVM: Support Vector Machine
